# White-nose syndrome pathology grading in Nearctic and Palearctic bats

**DOI:** 10.1371/journal.pone.0180435

**Published:** 2017-08-02

**Authors:** Jiri Pikula, Sybill K. Amelon, Hana Bandouchova, Tomáš Bartonička, Hana Berkova, Jiri Brichta, Sarah Hooper, Tomasz Kokurewicz, Miroslav Kolarik, Bernd Köllner, Veronika Kovacova, Petr Linhart, Vladimir Piacek, Gregory G. Turner, Jan Zukal, Natália Martínková

**Affiliations:** 1 Department of Ecology and Diseases of Game, Fish and Bees, University of Veterinary and Pharmaceutical Sciences Brno, Brno, Czech Republic; 2 CEITEC—Central European Institute of Technology, University of Veterinary and Pharmaceutical Sciences Brno, Brno, Czech Republic; 3 United States Department of Agriculture Forest Service, Northern Research Station, Columbia, Missouri, United States of America; 4 Department of Botany and Zoology, Masaryk University, Brno, Czech Republic; 5 Institute of Vertebrate Biology, Czech Academy of Sciences, Brno, Czech Republic; 6 Department of Veterinary Pathobiology, University of Missouri, Columbia, Missouri, United States of America; 7 Institute of Biology, Department of Vertebrate Ecology and Palaeontology, Wrocław University of Environmental and Life Sciences, Wrocław, Poland; 8 Laboratory of Fungal Genetics and Metabolism, Institute of Microbiology, Czech Academy of Sciences, Prague, Czech Republic; 9 Institute of Immunology, Friedrich-Loeffler-Institute, Federal Research Institute for Animal Health, Greifswald-Insel Riems, Germany; 10 Pennsylvania Game Commission, Harrisburg, Pennsylvania, United States of America; 11 Institute of Biostatistics and Analyses, Masaryk University, Brno, Czech Republic; Brown University, UNITED STATES

## Abstract

While white-nose syndrome (WNS) has decimated hibernating bat populations in the Nearctic, species from the Palearctic appear to cope better with the fungal skin infection causing WNS. This has encouraged multiple hypotheses on the mechanisms leading to differential survival of species exposed to the same pathogen. To facilitate intercontinental comparisons, we proposed a novel pathogenesis-based grading scheme consistent with WNS diagnosis histopathology criteria. UV light-guided collection was used to obtain single biopsies from Nearctic and Palearctic bat wing membranes non-lethally. The proposed scheme scores eleven grades associated with WNS on histopathology. Given weights reflective of grade severity, the sum of findings from an individual results in weighted cumulative WNS pathology score. The probability of finding fungal skin colonisation and single, multiple or confluent cupping erosions increased with increase in *Pseudogymnoascus destructans* load. Increasing fungal load mimicked progression of skin infection from epidermal surface colonisation to deep dermal invasion. Similarly, the number of UV-fluorescent lesions increased with increasing weighted cumulative WNS pathology score, demonstrating congruence between WNS-associated tissue damage and extent of UV fluorescence. In a case report, we demonstrated that UV-fluorescence disappears within two weeks of euthermy. Change in fluorescence was coupled with a reduction in weighted cumulative WNS pathology score, whereby both methods lost diagnostic utility. While weighted cumulative WNS pathology scores were greater in the Nearctic than Palearctic, values for Nearctic bats were within the range of those for Palearctic species. Accumulation of wing damage probably influences mortality in affected bats, as demonstrated by a fatal case of *Myotis daubentonii* with natural WNS infection and healing in *Myotis myotis*. The proposed semi-quantitative pathology score provided good agreement between experienced raters, showing it to be a powerful and widely applicable tool for defining WNS severity.

## Introduction

Wildlife conservation medicine is currently being challenged by a number of infectious and non-infectious diseases [1] that can potentially induce mass mortality events [2]. Recently, the health of temperate-region bats has been compromised by a generalist fungal agent that causes white-nose syndrome, *Pseudogymnoascus destructans* [3–7]. White-nose syndrome (WNS) emerged as a point-source epidemic. Its geographic spread since 2006 has been associated with a major decline in Nearctic bat populations [8–10]. On the other hand, Palearctic bat communities in Europe and Asia appear to tolerate hyperendemic exposure to this virulent pathogen [11].

WNS is characterised by the invasive skin infection caused by *P*. *destructans* [3,5,12,13]. Extensive damage to flight membranes may alter the torpor pattern of hibernating bats by increasing their arousal frequency and depleting their fat reserves prematurely [14,15]. Disruption of the effective skin’s barrier function can, therefore, explain the pathophysiological mechanisms underlying mortality in WNS-affected bats [16–20].

Pathognomonic skin lesions are the only reliable sign of this syndromic disease that are easy to detect using laboratory methods. In combination with identification of the pathogen [4,6], therefore, histopathology is seen as the gold standard for diagnosing WNS qualitatively [13]. While histopathology has indicated equivalent focal skin-tissue invasiveness in multiple bat species naturally infected with *P*. *destructans* throughout its known geographic range [7,11–13,21], it does not explain the striking difference in infection outcome between Nearctic and Palearctic bats [11]. Likewise, no significant difference in fungal load and cupping erosion size has been found on bats in Europe, Palearctic Asia and North America [11]. In order to better understand WNS progression and severity, quantification of histopathological findings in bats sampled from different regions is needed.

Until recently, bats had to be dead or euthanised for laboratory testing procedures, collection of dermato-histopathological samples from the wings, muzzle and ears and to optimise detection of the disease [13]. What is more, the severity scoring system for WNS currently in use utilises the whole membrane from one wing [14]. Both in Europe and elsewhere, bat species are under strict protection; hence, the need for a non-lethal sampling method is imperative. In response, our team recently validated a new non-lethal technique for identifying and targeting WNS skin lesions for sampling [22]. Trans-illumination of a wing membrane with 366–385 nm ultraviolet (UV) light elicits a distinct orange-yellow fluorescence that corresponds directly with the fungal cupping erosions in histological sections of the respective skin area. The fluorescence emitted from these skin lesions is associated with hyperaccumulation of riboflavin, a secondary fungal metabolite that may also represent a virulence factor leading to skin damage [23]. When not being used in combination with infected wing membrane biopsies, UV transillumination can also be used for non-invasive photographic surveillance of infection intensity.

Based on the need for a standardised non-lethal tool for measuring skin pathology in bats from different regions, the objective of the present study was to establish and validate a novel grading system for defining WNS severity based on single biopsies. Here, we propose a weighted cumulative WNS pathology score based on UV trans-illumination guided biopsies that allows for semi-quantitative comparison and shows high inter-pathologist reproducibility. We use this grading system to describe and compare histopathological features of *P*. *destructans* infection in both Nearctic and Palearctic bats previously qualitatively diagnosed with WNS. While non-lethal diagnostic tools offer the opportunity to follow the progression of skin pathology, prior experience with WNS pathology scores may be used to predict the outcome of infection in a diseased bat. With intensive research for treatment of WNS in North America [24], ability to predict patient prognosis becomes imperative. Lacking sufficient sample sizes for statistical model evaluation, case report experience might provide valuable information. We, therefore, used time series data on two bats receiving supportive care in a rehabilitation facility to document the clinical outcome of WNS and to examine the diagnostic utility of the proposed grading system in the early post-hibernation period.

## Material and methods

### Ethics statement

Collection of bat samples from hibernacula in the Czech Republic complied with Czech Law No. 114/1992 on Nature and Landscape Protection. Collection was based on permits 01662/MK/2012S/00775/MK/2012, 866/JS/2012 and 00356/KK/2008/AOPK issued by the Agency for Nature Conservation and Landscape Protection of the Czech Republic. Approval of all experimental procedures was provided by the Ethical Committee of the Czech Academy of Sciences (No. 169/2011). Sampling at the “Nietopierek” Natura 2000 site (Poland) was approved by the II Local Ethical Commission in Wrocław (No. 45/2015). Sampling in Latvia, Slovenia, Russia and Poland was approved by the Latvian Nature Conservation Agency (No. 3.15/146/2014-N), the Ministry of Environment and Spatial Planning of the Slovenian Republic, the Slovenian Environment Agency (No. 35601-35/2010-6), the Institute of Plant and Animal Ecology—Ural Division of the Russian Academy of Sciences (No. 16353–2115/325) and the Regional Directorate for Environmental Protection in Gorzow Wielkopolski (No. WPN-I-6205.10.2015.AI). Collection of samples from bats in Hannibal, MO complied with Missouri Department of Conservation Scientific Wildlife Collector’s Permit (No. 15947/2014) and was performed under a protocol approved by University of Missouri, Animal Care and Use Committee. The authors were authorised to handle wild bats according to the Czech Certificate of Competency (No. CZ01341; §17, Act No. 246/1992 Coll.) and a permit approved by the Latvian Nature Conservation Agency (No. 05/2014).

## Surveillance of bats for white-nose syndrome

The origin of Holarctic bat samples has been described previously [7,11,12]. Briefly, bats were sampled at 20 sites in the Czech Republic, Slovenia, Latvia, Poland, Russia and the USA. Additional Nearctic *Myotis septentrionalis* males were sampled in Hannibal, Missouri (39.70 N, 91.36 W; USA) from January to March 2015, two hibernation seasons after the first documentation of *P*. *destructans* infection.

As described previously [7,11,12], bat wings were swabbed for laboratory examination of *P*. *destructans* infection using culture and estimation of associated fungal load using quantitative polymerase chain reaction (qPCR), and photographed over a 368 nm UV lamp for later enumeration of lesions indicative of WNS [22]. One WNS-suspect spot from each bat was then sampled under UV guidance. In this way, we collected wing membrane biopsies from 210 specimens of 21 different Nearctic and Palearctic bat species during the late hibernation and early post-hibernation periods of 2012 to 2015 (S1 Table). All bats were handled in such a way as to minimise any impact from disturbance and then quickly released at the capture site. Skin samples collected with a 4 mm sterile punch (Kruuse, Denmark) were immediately fixed in 10% formalin, then dehydrated in the laboratory and embedded in paraffin. To obtain a representative range of histopathology findings, a series of 80 5 μm sections were prepared from each wing membrane biopsy and stained with periodic acid-Schiff (PAS). The slides were examined with light microscopy with focus on invasive fungal growth and identification of the WNS skin pathology grades described below.

### Case reports of WNS progression

In the Czech Republic, single specimens of *M*. *daubentonii* and *M*. *myotis* with extensive WNS infection were recognised at hibernacula in the Podyjí National Park and Jeseníky Mountains and sent to the Rescue Centre at the University of Veterinary and Pharmaceutical Sciences Brno (Czech Republic). While healing was being augmented with captive nutritional support, skin disease progression under euthermic conditions was documented using photography and histopathology.

### A novel grading system of WNS skin pathology

The semi-quantitative grading system presented here is based on a single bat flight membrane biopsy per animal guided by UV transillumination. Eleven binary encoded grades *g*, pathognomonic for WNS [13] or associated with the early stages of *P*. *destructans* skin infection, were selected as index signs for grading and were identified on Periodic acid-Schiff (PAS)-stained histopathological slides. The grades were ordered with increasing invasion severity from presence of the fungus on the wing’s surface to replacement of tissue by fungal hyphae throughout the wing’s thickness. Skin surface colonisation by the fungus (*g*_1_) was followed by hair follicle infection (*g*_2_), sebaceous gland infection (*g*_3_), single occurrences of cup-like lesions (*g*_4_), multiple and/or confluent cupping erosions (*g*_5_), skin basement membrane breach by the fungus (*g*_6_) and full-thickness invasion (*g*_7_). The bat’s immune response to infection was represented by variable inflammatory response manifested as tissue infiltration with neutrophils (*g*_8_) and fungal sequestration by extensive inflammatory response (*g*_11_). Findings of skin necrosis (*g*_9_), characterised by loss of identifiable skin structures and skin infarction (*g*_10_), were also included among the criteria associated with *P*. *destructans* skin infection.

In order to evaluate infection severity, we assign a weight *w* to each grade based on its invasiveness and extensiveness. The weights were selected so that a cumulative score of lower grade findings could not outweigh more severe grades of skin infection. A combination of fungal skin colonisation (*w*_1_ = 1) and hair follicle (*w*_2_ = 2) and sebaceous gland infection (*w*_3_ = 2) represent the least severe finding, with a cumulative score lower than that in samples with single (*w*_4_ = 6) or multiple (*w*_5_ = 12) cupping erosions. Basement membrane breach (*w*_6_ = 13) and full thickness infection (*w*_7_ = 19) represent severe disruptions of the deep layers of bat wing membranes. We consider inflammation (*w*_8_ = 20), necrosis (*w*_9_ = 25) and infarction (*w*_10_ = 30) to be the most severe WNS grades. When the immune response succeeds in fungal sequestration (*w*_11_ = −20 to reflect potential healing), however, recovery from WNS appears more likely.

Inspection of 80 wing membrane sections from each biopsy could result in a weighted cumulative WNS pathology score defined as:

$$histoSum=\sum_{i=1}^{11}g_{i}w_{i},$$

where *g* ∈ {0,1}, with *g* = 0 meaning absence and *g* = 1 meaning presence of the respective WNS pathology grade. The histoSum values may range from −20 (binary code corresponding to the listed grades: 00000000001) to 130 (11111111110). As an example, a sample scored 32 if the biopsy contained fungal skin colonisation (+1) together with multiple cupping erosions (+12) and hyphae that breached the basal membrane (+13). Note that a single cupping erosion (+6) is scored automatically when multiple or confluent cupping erosions are present.

Data used in this study is available in S2 Table.

### Inter-pathologist reproducibility of the proposed grading system

In order to test whether the above-described index signs for WNS grading were universally recognizable, we photographed 30 randomly selected PAS-stained sections prepared from bat-wing biopsies. In a blind evaluation, five independent pathologists examined and scored the photographs. Clarity of grading in naïve raters was evaluated in an experiment whereby 72 undergraduate students from the University of Veterinary and Pharmaceutical Sciences Brno scored a subset of 10 photographs following 45-minutes training in WNS histopathology. The participating students of veterinary medicine already received education in general pathology during their veterinary study program and volunteered for the task of scoring *P*. *destructans* skin infection pathology. These volunteers were recruited at a Wildlife Diseases lecture. Assignment to three evaluating groups was performed to limit the time that the volunteers spent reading WNS histopathology.

### Estimation of fungal load

Fungal DNA was isolated from swabs of the dorsal side of bat left wing using QIAamp DNA Mini Kit (Qiagen, Halden, Germany). DNA from *P*. *destructans* was quantified with a dual-probe TaqMan (Life Technologies, Foster City, CA, USA) assay developed by Shuey et al. [25], following the detailed protocol for the qPCR of Zukal et al. [11]. Each sample was run in triplicate and each plate included positive and negative controls. Fungal load was estimated from positive control dilution series calibration curve according to equation log (*P*. *destructans* DNA concentration) = 3.194–0.287 * cycle, and standardized to total fungal load in nanograms per 1 cm^2^ of wing area to correct for species size differences [11].

### Statistical analyses

Weighted cumulative WNS grading scores for the Palearctic and Nearctic zones were compared using the Mann-Whitney *U* test. The influence of species-specific histoSum was assessed by filtering the value for species random effect using the *lme4* package in R [26,27].

The relationship between data provided by non-destructive diagnostic methods and initial progression of WNS infection by histopathology was evaluated using logistic regression [27]. Two sets of three logistic regressions were fitted for skin surface colonisation by the fungus, formation of single and multiple cupping erosions. Fungal load (log_10_(ng)) and number of UV-fluorescent spots on the left wing recalculated to cm^2^ of wing membrane [11] were used as independent variables in the respective models. Wing membrane area for *M*. *septentrionalis* was not available in literature and thus it was calculated from UV photographs using a custom R script and the *splancs* and *jpeg* packages [28,29]. The relationship between histoSum and number of UV fluorescent spots was evaluated using phylogenetic generalised least-squares [30] in the *phytools* package in R [31]. Previously published multilocus phylogeny was used to correct for species relatedness [7].

Inter-rater agreement of pathologists and veterinary students was tested in a paired study using the unweighted Cohen’s *κ* coefficient [32] against scores included in the results below, where confidence was estimated with equations from [33]. The overall evaluation of *κ* for multiple raters was used according to Fleiss et al.’s modification [34]. The *κ* coefficient corrects grading category agreement frequency for an effect where raters achieve consensus by chance. We used custom R scripts along with the *irr* package [35]. Confidence intervals for multi-rater agreement were estimated from 1000 bootstrap replicates.

## Results

**Fig 1:**
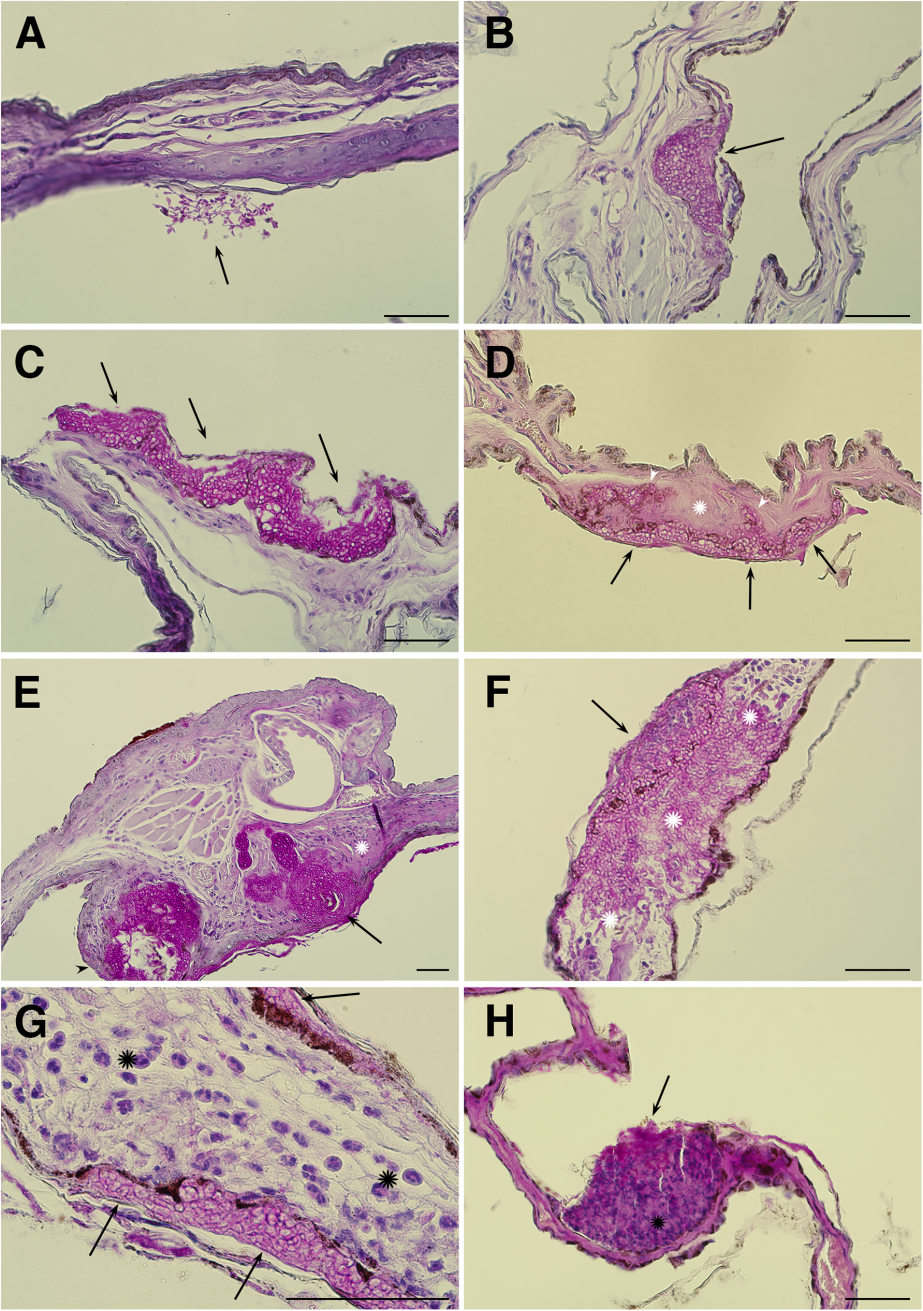
Histopathology grades induced by natural *Pseudogymnoascus destructans* skin infection in Holarctic bats. (A) *Myotis myotis*: fungal skin-surface colonisation with aerial hyphae developing conidia (*g*_1_, black arrow), not classified as WNS in absence of other findings; (B) *M*. *myotis*: a single cupping erosion (*g*_4_, black arrow) eroding to the epidermal/dermal interface; (C) *M*. *myotis*: three confluent cupping erosions (*g*_5_, black arrows); (D) *M*. *daubentonii*: necrotic wing membrane (witnessed as loss of dermal tissue stainability; *g*_9_, white asterisk) next to multiple cupping erosions packed with *P*. *destructans* hyphae (*g*_5_, black arrows) that also breached the basement membrane (*g*_6_, white arrowheads); (E) *M*. *daubentonii*: infection of hair follicle (*g*_2_, black arrow) and associated glands (*g*_3_, black arrowhead). Surface skin colonisation (*g*_1_), multiple cupping erosions (*g*_5_), inflammatory cells (*g*_8_) and necrotic tissue (*g*_9_, white asterisk) are also present in the section; (F) *M*. *dasycneme*: an outline of a cupping erosion (*g*_4_, black arrow) clearly visible together with full thickness fungal invasion (*g*_7_) replacing the necrotic wing membrane (*g*_9_) and sporadic neutrophils (*g*_8_, white asterisk); (G) *M*. *lucifugus*: marked inflammatory response (*g*_8_, black asterisks) to fungal invasion of several cupping erosions (*g*_5_, black arrows) on both sides of the wing membrane; (H) *M*. *dasycneme*: fungal sequestration (*g*_11_, black arrow) with neutrophils (*g*_8_, black asterisk) from the wing membrane. Periodic acid-Schiff stain. Scale bar– 50 μm.

**Fig 2:**
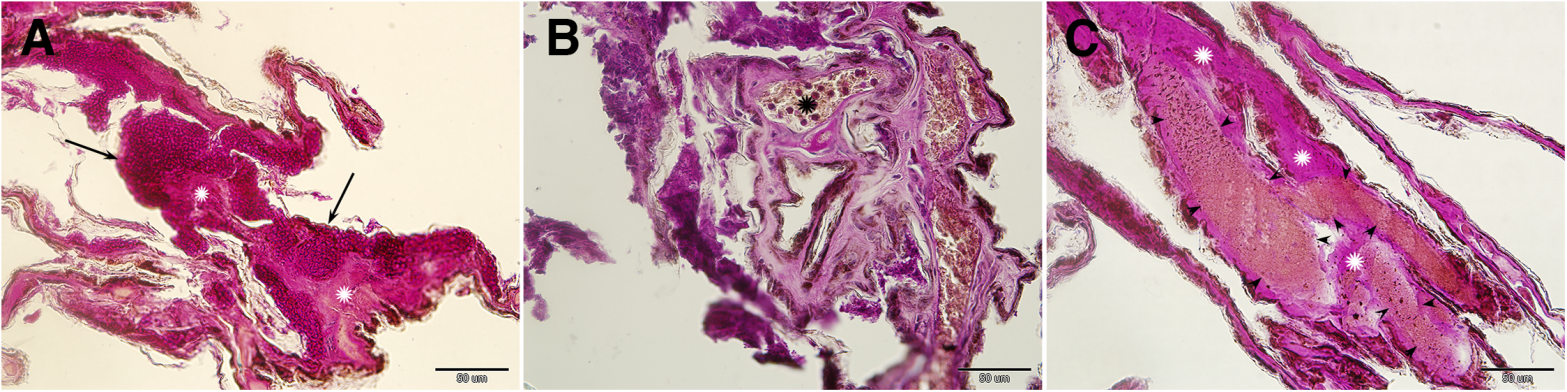
Skin infarction and necrosis associated with progressive white-nose syndrome lesions in *Myotis daubentonii*. Samples for histopathology were collected on Day 7 of the time series documented using UV and daylight photography. Extensive *Pseudogymnoascus destructans* infection of the wing membrane produced confluent cupping erosions (*g*_5_, black arrows) resulting in skin necrosis (*g*_9_, white asterisk), characterised as loss of identifiable skin structures (A). Intraluminal neutrophilic infiltration (*g*_8_, black asterisk) in distended blood vessels was associated with the compromised wing membrane (B). Other skin lesions in this bat included haemorrhagic infarcts (*g*_10_, black arrowhead) with stagnant blood and skin necrosis (*g*_9_, white asterisk) (C). Periodic acid-Schiff stain.

Prevalence of each WNS pathology grade (Figs 1 and 2) induced by natural *P*. *destructans* skin infection in Palearctic and Nearctic bat species varied from 0 to 100% in different bat species (Table 1). Signs of fungal skin-surface colonisation not classified as WNS (in the absence of other lesions) were present in all Nearctic bats and in 89% of Palearctic bats. Prevalence of infection within hair follicles and associated glands was highly variable in all hibernating bat species and for species with multiple investigated individuals ranged from 17 to 67% for the hair follicle infection and from 15 to 67% for the sebaceous gland infection. While wing membrane infection progressed to multiple and/or confluent cupping erosions in most bat species, single cupping erosions only were observed in *Miniopterus schreibersii* (*n* = 1), *M*. *bechsteinii* (*n* = 3) and *Rhinolophus euryale* (*n* = 1). Fungal hyphae regularly breached the epidermal/dermal interface in *M*. *lucifugus* (100%, *n* = 10) and *M*. *septentrionalis* (86%, *n* = 7) as well as in Palearctic bats (46–100%). Full thickness invasion of the wing membrane occurred more frequently in Palearctic bats (34% on average) and *M*. *septentrionalis* (71%) than in *M*. *lucifugus* (30%). A high percentage of both Palearctic and Nearctic bats (73 and 76% on average, respectively) showed an inflammatory response to fungal invasion, though this mostly resulted in a considerably lower occurrence of fungal sequestration (25 and 22% on average, respectively; Table 1). While *Eptesicus serotinus* (*n* = 1), *M*. *alcathoe* (*n* = 7), *Nyctalus noctula* (*n* = 8), *Pipistrellus pipistrellus* (*n* = 2), *Pipistrellus pygmaeus* (*n* = 2) and *Plecotus austriacus* (*n* = 1) were also sampled for histopathology, all were confirmed negative for WNS. Among these, four *M*. *alcathoe* were confirmed positive for *P*. *destructans* infection using qPCR (S2 Table).

**Table 1:** Prevalence of histopathology severity grades associated with natural skin infection by *Pseudogymnoascus destructans*. Prevalence was calculated as the percentage of positive biopsies at the given grade in the dataset of all bats positive for WNS based on histopathology. All infection grades apply to the wing membrane and, aside from fungal skin surface colonisation in the absence of any other findings, are classified as white-nose syndrome (WNS). Tested = number of individuals examined for histopathology. WNS histo+ = number of individuals confirmed positive for WNS. Std. error (%) = $100\sqrt{p(1-p)/n}$, where *n* is the overall number of scored grades for the given species and *p* is the proportion of positively scored grades. The respective severity grades are shown in Figs 1 and 2. Numbers in brackets represent WNS pathology grade weighting—see text for details.

Bat taxon data	WNS histo +	Std. error (%)	Prevalence (%)										
			Fungal skin colonisation (1)	Hair follicle infection (2)	Sebaceous gland infection (2)	Single cupping erosion (6)	Multiple cupping erosions (12)	Basement membrane breach (13)	Full thickness infection (19)	Inflammation (20)	Fungal sequestration (−20)	Necrosis (25)	Infarction (30)
*Barbastella barbastellus*	3	8.67	100	0	0	100	100	100	66.67	66.67	0	66.67	0
*Eptesicus nilssonii*	3	7.75	100	66.67	66.67	100	100	100	66.67	100	33.33	66.67	0
*Miniopterus schreibersii*	1	14.5	100	100	100	100	0	100	0	100	0	100	0
*Myotis bechsteinii*	3	8.21	66.67	33.33	0	100	0	66.67	0	66.67	0	33.33	0
*Myotis brandtii*	1	8.67	100	100	100	100	100	100	100	100	100	100	0
*Myotis dasycneme*	13	4.18	100	30.77	15.38	100	38.46	61.54	23.08	84.62	30.77	38.46	0
*Myotis daubentonii*	10	4.76	90	30	20	100	70	70	50	50	10	70	10
*Myotis emarginatus*	6	6.15	100	16.67	16.67	100	83.33	66.67	50	50	16.67	50	0
*Myotis myotis*	50	2.09	96	46	36	100	46	46	2	34	6	24	0
*Myotis nattereri*	4	7.02	100	25	25	100	25	50	0	25	0	0	0
*Plecotus auritus*	7	5.69	100	42.86	28.57	100	71.43	71.43	28.57	71.43	28.57	42.86	0
*Rhinolophus euryale*	1	14.5	0	0	0	100	0	100	0	100	100	0	0
*Rhinolophus hipposideros*	4	7.25	100	50	50	100	75	100	50	100	0	75	0
**Total/mean in Palearctic bats**	106		88.67	41.64	35.25	100	54.56	79.41	33.61	72.95	25.03	51.31	0.77
**Std. error in Palearctic bats**			2.06	4.75	4.46	0.0	4.85	4.75	3.87	4.85	4.71	0.94	3.29
*Myotis lucifugus*	10	4.7	100	30	30	100	100	100	30	80	30	40	0
*Myotis septentrionalis*	7	5.62	100	14.29	14.29	100	100	85.71	71.43	71.43	14.29	71.43	0
**Total/mean in Nearctic bats**	17		100	22.14	22.14	100	100	92.86	50.71	75.72	22.14	55.72	0
**Std. error in Nearctic bats**			0.0	10.29	10.29	0.0	0.0	5.71	12.11	10.29	12.11	0.0	10.29

### Sum of scores and inter-pathologist agreement in the proposed grading system

**Fig 3:**
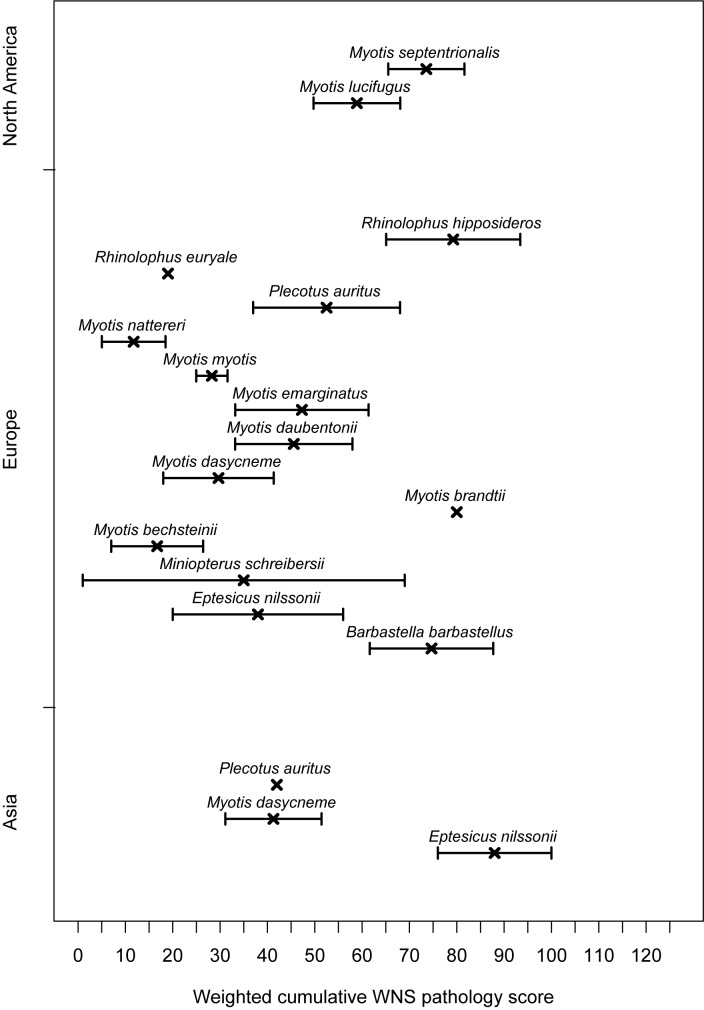
Species-specific weighted cumulative white-nose syndrome pathology score (histoSum). Average sum of weighted qualitative scoring for white-nose syndrome severity grades displayed in Figs 1 and 2 (± std. error). Animals with histoSum = 1 not classified as positive for WNS on histopathology are included in the figure. Species sampled on multiple continents are presented separately. See Table 1 for sample sizes.

While the histoSum score can reach a theoretical maximum of 130, the maximum score in practice was 126 for a fatal case of WNS in the *M*. *daubentonii* specimen described below (binary code 10011111011). The species with highest average histoSum were *Eptesicus nilssonii* from Asia (*n* = 2), *M*. *brandtii* (*n* = 1), *Rhinolophus hipposideros* (*n* = 4), *Barbastella barbastellus* (*n* = 3) from Europe and *M*. *septentrionalis* from North America (*n* = 7; Fig 3). While the mean histoSum for all sampled bats was 39.2 (std. error = 2.74), *E*. *nilssonii* (*n* = 2), *M*. *schreibersii* (*n* = 2), *M*. *bechsteinii* (*n* = 7), *M*. *dasycneme* (*n* = 6), *M*. *myotis* (*n* = 57), *M*. *nattereri* (*n* = 8) and *R*. *euryale* (*n* = 1) from Europe had a lower average weighted sum of concurrent findings (Fig 3). Nearctic bats had a significantly higher histoSum score (mean ± std. error: 64.9 ± 6.4) than Palearctic bats (35.9 ± 2.9; Mann-Whitney *U* = 1688, *p* < 0.001). Correction for random effect of species had no influence on significance.

Paired scoring agreement corrected for chance between five experienced pathologists and results presented herein ranged from 0.76 to 0.85 (Cohen’s *κ*, *p* < 0.001; 95% confidence intervals (CI): {[0.75, 0.87], [0.8, 0.91], [0.72, 0.85], [0.77, 0.89], [0.7, 0.83]}) with an overall Fleiss’ *κ*_5_ of 0.78 (*z* = 57.49, *p* < 0.001, CI: [0.74, 0.82]), signifying good agreement in scoring WNS severity. Inter-rater agreement evaluating each WNS pathology grade separately differed, with basement membrane breach and inflammation being most difficult to score consistently (Table 2). Naïve raters displayed lower inter-rater agreement with the reported scores (Cohen’s *κ* ∈ [0.5, 0.92]) and lower within-group agreement (Fleiss’ *κ* ∈ [0.64, 0.71]).

**Table 2:** Inter-rater agreement in scoring white-nose syndrome (WNS) pathology according to Fleiss’ *κ*. Experienced pathologists (*n* = 5) scored 30 photographs of randomly drawn histopathology slides, each group of naïve raters (*n* ∈{27, 20, 25}) scored a subset of 10 photographs. Standard errors for the given sample sizes were 0.047, 0.017, 0.023 and 0.018, respectively. Negative *κ* values indicate no inter-rater agreement.

Grade index	WNS pathology grade	Fleiss’ *κ*			
		experienced	naïve group 1	naïve group 2	naïve group 3
1	Fungal skin colonisation	0.671	0.560	0.114	0.014
2	Hair follicle infection	0.931	0.690	0.772	0.850
3	Sebaceous gland infection	0.712	0.074	0.575	0.839
4	Single cupping erosion	0.790	0.521	0.200	0.655
5	Multiple cupping erosions	0.861	0.716	0.445	0.596
6	Basement membrane breach	0.496	0.592	0.237	0.285
7	Full thickness infection	0.704	0.655	0.330	0.809
8	Inflammation	0.469	0.436	0.368	0.392
9	Skin necrosis	0.460	0.293	0.414	0.288
10	Skin infarction	0.851	-0.007	0.415	-0.008
11	Fungal sequestration	0.805	0.755	-0.032	0.159

### Progression of severe WNS skin infection resulting in extensive skin necrosis and fatality in a *Myotis daubentonii* bat

**Fig 4:**
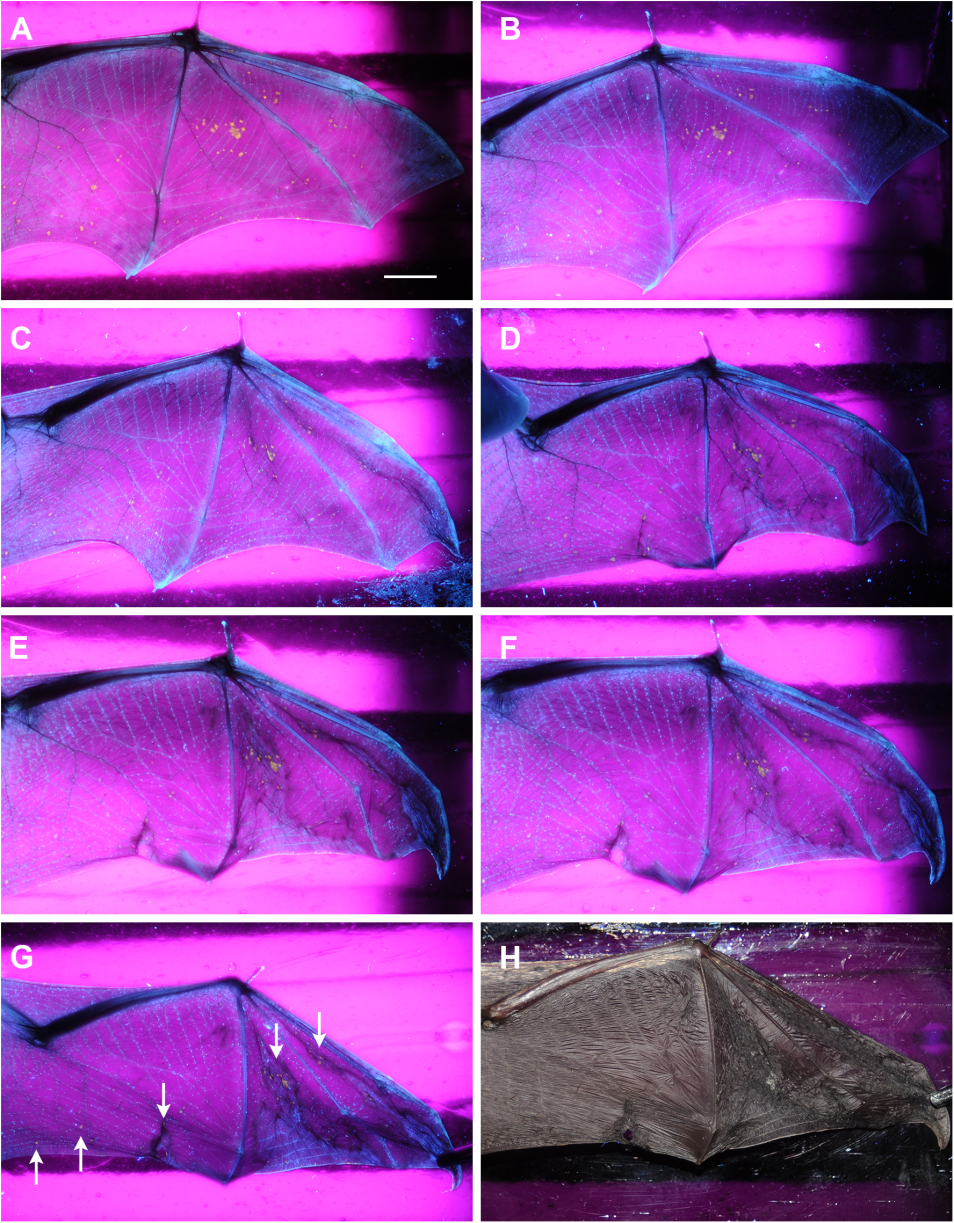
Time series of a *Myotis daubentonii* wing showing progression of white-nose syndrome lesions to fatality. Extensive white-nose syndrome infection was recognised on a *M*. *daubentonii* bat at a hibernaculum in the Podyjí National Park (Czech Republic). The bat was kept under euthermic conditions at a rescue centre, fed *ad libitum* and supplied with drinking water. The wing was extended over a Wood’s lamp at 366 nm wavelength and photographed in a darkroom. (A) = Day 0, (B) = Day 1, (C) = Day 2, (D) = Day 3, (E) = Day 5, (F) = Day 6, (G) = Day 7, white arrows indicate biopsy punch sites (results presented in Fig 5). Day 7 was also documented using daylight photography (H). Scale bar = 1 cm. This time series spanned seven days from capture at the hibernaculum to death in the rescue centre. Wing membrane areas with extensive *Pseudogymnoascus destructans* infection became dry and necrotic within two days of euthermy, whereupon they contracted and tore around the white-nose syndrome lesions in a proximal-to-distal pattern. The animal displayed loss of skin tone, elasticity and surface sheen, and ceased eating one day prior to death.

Progression of severe WNS lesions on the wing membranes of a *M*. *daubentonii* specimen was investigated at the University of Veterinary and Pharmaceutical Sciences Brno (Czech Republic) Rescue Centre. A time series of UV transillumination and daylight photography images spanning seven days from capture to death indicated that the wing membranes started to show signs of dry necrosis within two days (Fig 4). Flight membrane areas with extensive infection lost tone, elasticity and sheen as they contracted and tore around the WNS lesions in a proximal-to-distal pattern. Histopathology demonstrated wing membrane necrosis associated with confluent cupping-erosion from fungal infection, distended blood vessels with intraluminal neutrophilic infiltration and haemorrhagic infarct consisting of red blood cell and fibrin clots caused by blood flow obstruction (Fig 2).

### Healing time series of WNS lesions in a *Myotis myotis* bat

**Fig 5:**
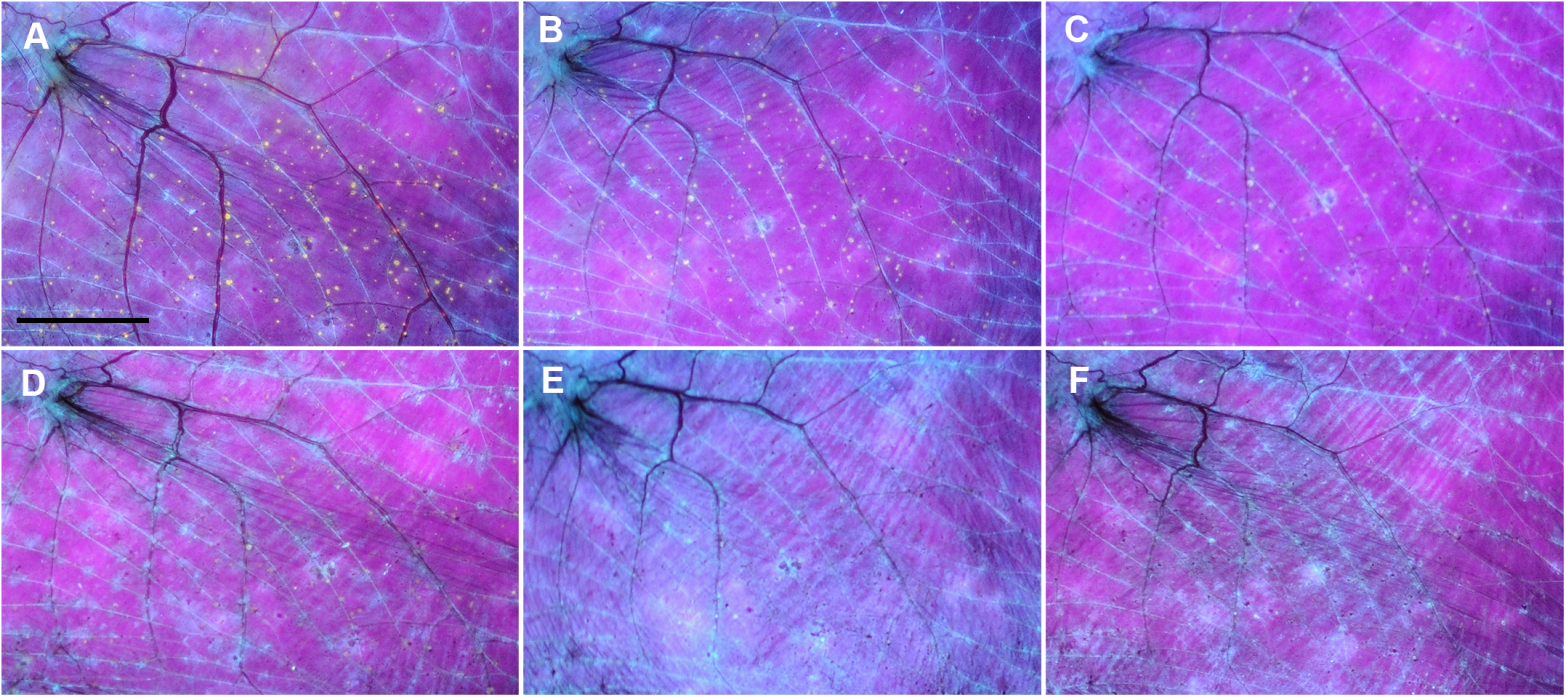
UV transillumination time series of a *Myotis myotis* wing with decrease in fluorescence corresponding to white-nose syndrome lesions over time. The bat was captured at the end of the hibernation period, kept in captivity at euthermy, fed *ad libitum* with cockroaches and mealworms and supplied with drinking water, and released after the white-nose syndrome lesions had healed. The wing was extended over a Wood’s lamp at 366 nm wavelength and photographed in a darkroom. **A** = Day 0, **B** = Day 3, **C** = Day 5, **D** = Day 7, **E** = Day 11, **F** = Day 15. The top-left corner matches in each image, wing deformation is due to variable handling of the live animal during sampling. Scale bar = 1 cm.

**Fig 6:**
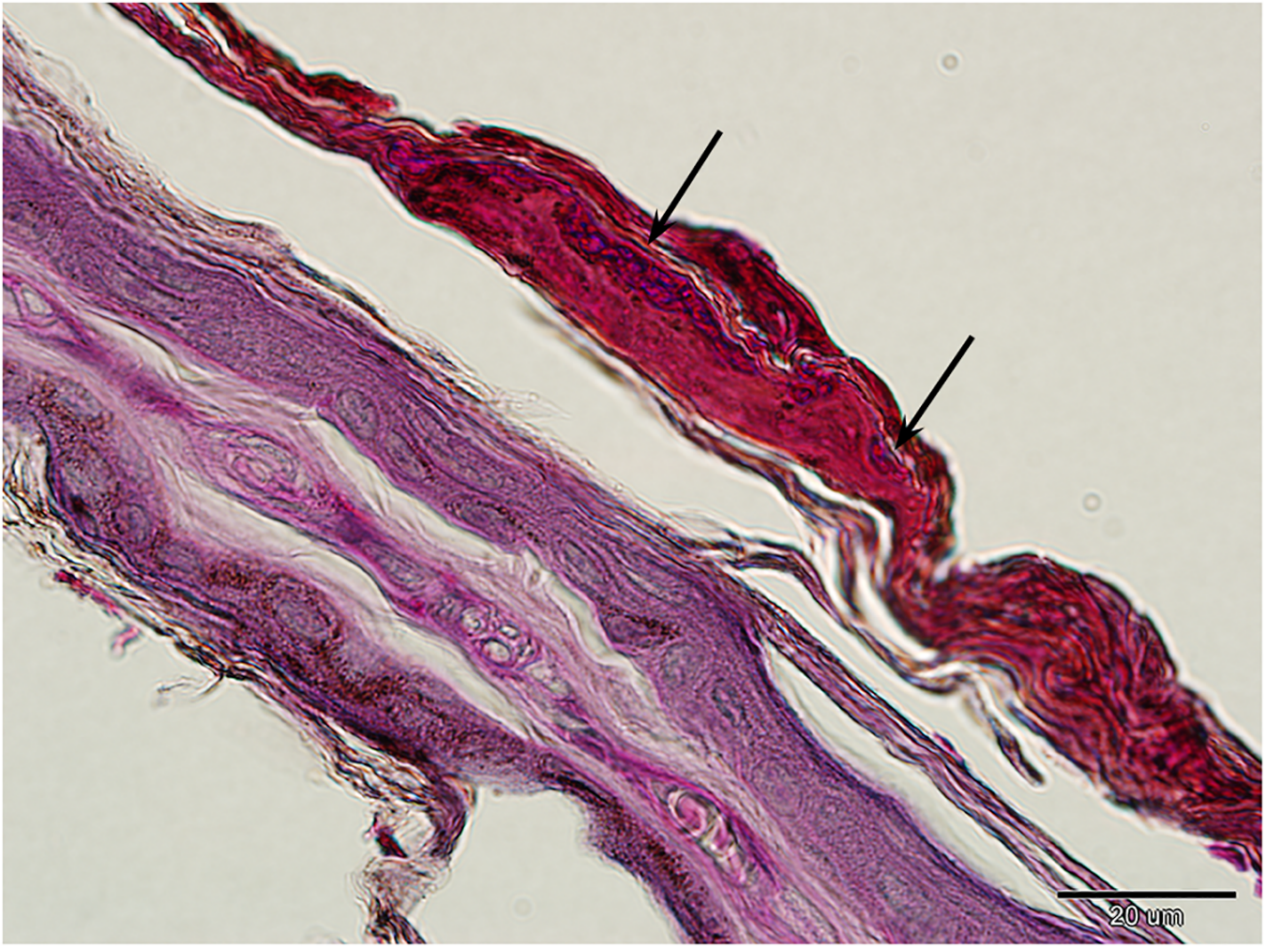
Healing of a white-nose syndrome lesion in *Myotis myotis*. Samples for histopathology were collected on Day 15 of the time series described in Fig 5. A cupping erosion-like structure (black arrow) packed with fungal hyphae within a scab covering the healing wing membrane of *M*. *myotis*. Periodic acid-Schiff stain.

A *M*. *myotis* bat captured at the end of the hibernation period was diagnosed with pathognomonic WNS skin lesions using UV transillumination (Fig 5A) and histopathology (Fig 1B). The fluorescent yellow-orange WNS lesions disappeared after two weeks at euthermy (Fig 5). Healing progressed until cupping erosion-like structures packed with fungal hyphae were contained within a scab covering the repaired wing membrane (Fig 6). Though this specimen had a histoSum score of 31 (11110001000) on the day of capture, the score had decreased to -20 (00000000001) after 15 days of healing at euthermy.

### Functional analysis of WNS pathology scores

**Fig 7:**
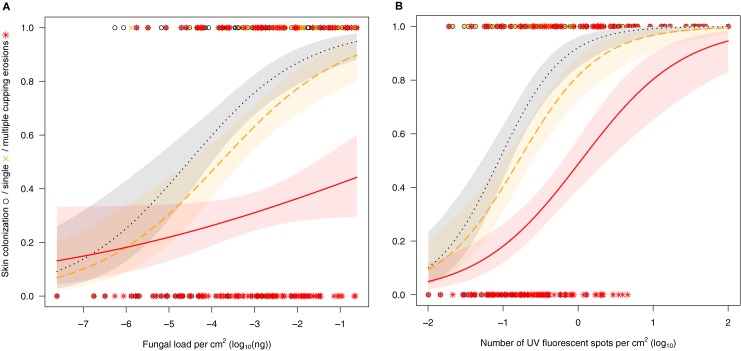
Relationship between data from non-destructive diagnostic methods and selected histopathology severity grades. Logistic regressions of skin surface colonisation by the fungus (black circle, dotted line; coefficients: (A) *β*_0_ = 3.38, *β*_1_ = 0.74, (B) *β*_0_ = 2.45, *β*_1_ = 2.32), single cupping erosion (orange cross, dashed line; coefficients: (A) *β*_0_ = 2.60, *β*_1_ = 0.68, (B) *β*_0_ = 1.49, *β*_1_ = 1.88) and multiple cupping erosions (red star, solid line; coefficients: (A) *β*_0_ = -0.09, *β*_1_ = 0.24, (B) *β*_0_ = -0.06, *β*_1_ = 1.44) dependent on fungal load detected on qPCR in Palearctic bats (A) or number of UV fluorescent spots in Holarctic bats (B). Shaded area represents 95% confidence interval on predicted probability.

Logistic regression (Fig 7A) revealed that the odds of observing surface skin colonization on histopathology increase with increase in fungal load estimated from wing swabs of Palearctic bats (odds ratio: *OR* = 2.1, CI: 1.6–2.8, *p* < 0.001; fungal load was not available for histologically examined Nearctic bats, S2 Table). The *OR* for observing single cupping erosions dependent on fungal load is lower, but significantly different from 1 (*OR* = 2.0, CI: 1.5–2.6, *p* < 0.001), indicating that single cupping erosions occur in response to roughly one-order-of-magnitude higher fungal loads than that during fungal skin-surface colonisation (Fig 7). In Palearctic bats, wing membrane infection progressed to multiple and/or confluent cupping erosions with fungal loads randomly (*OR* = 1.3, CI: 1–1.6, *p* = 0.54). Investigating both Palearctic and Nearctic bats (S2 Table), a similar pattern was observed in odds of recognizing surface skin colonization (*OR* = 10.2, CI: 4.7–22.2, *p* < 0.001) and single cupping erosions (*OR* = 6.6, CI: 3.5–12.5, *p* < 0.001) on histopathology dependent on the number of UV fluorescent lesions (Fig 7B). The number of UV fluorescent lesions was a better predictor for multiple and/or confluent cupping erosions with *OR* = 4.3 (CI: 2.6–7.2, *p* < 0.001) than fungal load.

**Fig 8:**
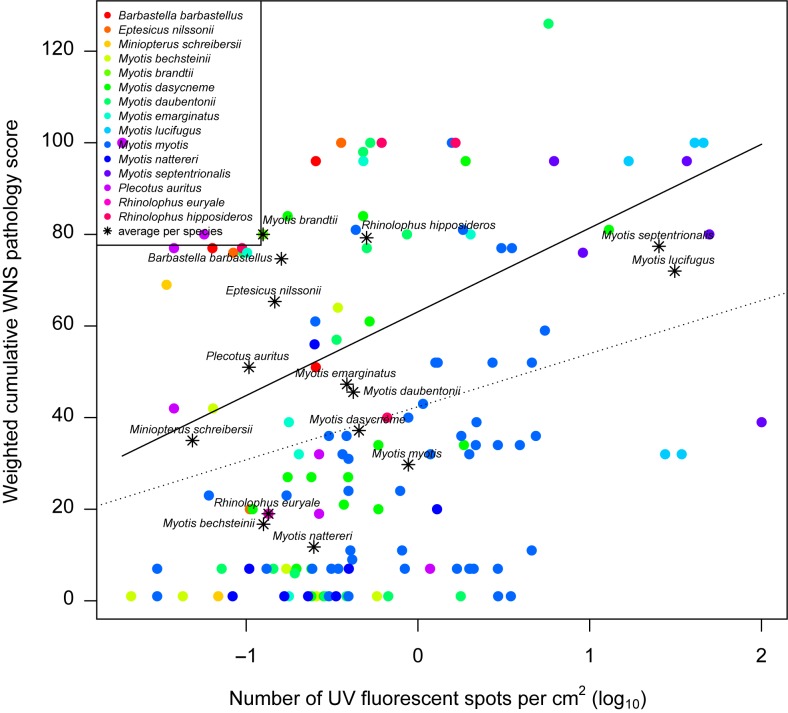
Increase in weighted cumulative white-nose syndrome pathology score with progressing UV fluorescence. Phylogenetic generalised least-squares accounts for phylogeny and intraspecific variability in evaluating the relationship between weighted cumulative white-nose syndrome pathology score dependent on unit number of UV fluorescent lesions (*β*_0_ = 62.1, *β*_1_ = 16.41; solid line). Linear regression without phylogenetic correction (*α* = 42.38, *β* = 11.59, *F*_1,131_ = 9.12, *p* = 0.003, *r*^2^ = 0.07; dotted line).

Cumulative WNS pathology scores from single 4 mm biopsies increased with the number of UV fluorescent lesions observed on the surface of the whole wing (*β*_0_ = 62.1, *β*_1_ = 16.41; Fig 8), indicating that species with more extensive UV fluorescence tend toward multiple concurrent WNS findings on histopathology.

## Discussion

Fuelled by no reports of mass die-offs from Europe [36,37], the most erroneous conclusion concerning white-nose syndrome is the belief that *P*. *destructans* infection causes no harm to Palearctic bats. *Pseudogymnoascus destructans* infection is known to induce complex physiological and transcriptional effects in hibernating bats long before the onset of the clinical signs indicative of late-stage WNS [19,38]. Palearctic and Nearctic bats are exposed to similar fungal loads, resulting in equivalent focal skin-tissue invasiveness pathognomonic for WNS lesions [7,11–13,39,40]. It would be reasonable, therefore, to expect both morbidity effects (not always recognisable in the field) and mortality across the distribution range of *P*. *destructans*. Here, we show the full range of WNS skin pathology in Palearctic and Nearctic bats. Moreover, we document a case of *P*. *destructans* infection in a European *M*. *daubentonii* bat progressing to fatality due to extensive skin necrosis and infarction. The differential outcome of WNS in Eurasia and North America appears to be associated with some form of tolerance mechanism [11]. Our data, however, suggests that Palearctic bat tolerance to WNS infection may be limited by severity of wing damage.

In this paper, we presented a novel pathogenesis-based grading system for *P*. *destructans* skin infection that utilises non-lethal biopsy sampling to examine the WNS lesions resulting from host-pathogen interaction. The severity grades chosen are simple to differentiate on histopathology and show good agreement amongst experienced raters. Identification of inflammation and skin necrosis exhibited lowest agreement and requires careful consideration in multi-rater comparisons. Naïve, minimally trained, raters showed similar agreement with scoring most grades as did experienced raters, but fungal skin colonization, sebaceous gland infection, skin infarction and fungal sequestration were most challenging grades for this group (Table 2). Generally speaking, histopathology enables one to assign nominal diagnostic categories to medical conditions observed in organs and tissues. Grading may provide additional data allowing one to estimate severity, predict biological behaviour of the disease, predict survival rate and make decisions regarding patient prognosis and treatment [41]. Recent studies linking histopathology WNS severity scores with frequent arousal from hibernation and mortality [14] and altered physiological homeostasis [18–20,42] have used destructive methods for examining bats. While such methods for examining wing membrane damage score WNS severity only at the moment of death or euthanasia [14,18–20], our grading system allows one to follow temporal patterns of disease progression with consecutive multiple biopsies targeted with UV transillumination. UV fluorescence-guided non-lethal biopsy diagnostics of WNS skin showed 95.5% sensitivity and 100% specificity [22]. Collection of additional biopsies from each specimen may further increase diagnostic and grading sensitivity.

Direct comparison between histological WNS severity scoring from the whole wing membrane, which provides a reasonable representation of skin damage associated with *P*. *destructans* infection [14,20], and our proposed single non-lethal biopsy method is complicated. Instead, we suggest that the number of bats positive for WNS lesions on histopathology (*n* = 123) and the number of taxa (*n* = 15) from the Palearctic and Nearctic regions examined, together with the non-lethal nature of the methodology, make this grading system universally applicable to all species exposed to the infection. The ability to select a biopsy site on the bat’s wing displaying the most representative and extensive UV fluorescence [22] enables a reasonable assessment of pathology while reducing the impact of WNS surveillance on the bat.

Previous WNS grading schemes have proposed four or five WNS-positive grades [14,20], with presence, extent and distribution of skin lesions densely packed with fungal hyphae (cupping erosions) used as the main criteria for assigning whole-wing-based scores [14]. As an alternative, we propose a finer, semi-quantitative 11-grade scale. Analogous quantitative measures of *P*. *destructans* skin infection in this alternative grading system include presence of single, multiple and/or confluent cupping erosions within a 4 mm punch biopsy that can be evaluated through image analysis to address invasiveness and size [11], then approximated for the whole wing from UV photographs [22]. Expanding the previous systems towards more severe pathology means that invasive fungal growth penetrating the full-thickness of the wing membrane represents a higher grade of skin infection than cup-like fungal structures eroding to the epidermal/dermal interface only [12,13]. In addition to cupping erosions, the ability of *P*. *destructans* to invade the dermis and the full thickness of the wing membrane distinguishes WNS from other dermatophytic infections.

As dysregulated immunity may contribute to severe post-emergent pathology [43], we also included bat immune response to infection (inflammation and fungal sequestration) as index criteria in our grading system. Inflammatory responses were frequently associated with fungal invasion in both Palearctic and Nearctic bats, probably due to sampling in the late-hibernation or early post-hibernation periods. However, the ability of bats to contain the infection through sequestration of the fungus with neutrophils and skin debris was only observed in a quarter of bats showing an inflammatory response. Warnecke et al. [20] scored bat tissue replaced by the fungus as necrotic. Unlike their indirect indication, we considered skin necrosis as findings characterised by loss of staining pattern, indistinct outlines of individual bat tissue cells and hypereosinophilia (Figs 1D and 2A). Skin infarction, which has previously been recognised in association with WNS [16], accounted for the most severe wing membrane damage (Fig 2C). In future studies, therefore, we urge that WNS severity be assessed with respect to skin infarction, as wing membrane damage in such cases extended from WNS skin lesions to terminal wing areas affected with blood flow obstruction (cf. Fig 4).

While some hibernating bats are able to recover from fungal infection [44,45], extensive skin damage in WNS survivors may alter flight performance, foraging efficiency and metabolism during the post-emergent season [16,43,46,47]. In line with the ecological and evolutionary trade-off concept [48], mounting an immune response against *P*. *destructans* infection, and the necessity to repair the damaged wing membrane, represent physiological costs that may affect the bat’s fitness and survival. To address the series of healing from different grades of skin damage associated with WNS, our histopathology findings in conjunction with the case reports suggest that cupping erosions heal through marked neutrophilic inflammation (Fig 1G), sequestration of the agent from the skin (Fig 1H) and re-epithelialization (Figs 1H and 6). As shown in Fig 6, the outcome of healing is regeneration of the skin, suggesting a return to full function. On the other hand, tissue necrosis (Figs 1D and 2A) and full-thickness skin infection (Fig 1F) probably need more remodelling and may result in wing membrane scarring. Haemorrhagic infarcts (Fig 2C) induce larger flight membrane defects (Fig 4) and may result in tears that stretch to the wing margin (Fig 4E, 4F, 4G and 4H).

Case reports on infection time-series in *M*. *daubentonii* and *M*. *myotis* document the dichotomy of development that may occur in European bats infected with *P*. *destructans*. Our findings indicate that the severity of invasion and associated wing damage determine the clinical outcome in terms of morbidity and/or mortality. Importantly, the *M*. *daubentonii* in our case study died during the early post-hibernation period, similar to observations by Meteyer et al. [43]. Sporadic cases of mortality in European bats due to *P*. *destructans* infection may go undetected, therefore, outside of hibernacula. From a practical diagnostic point of view, it is necessary to bear in mind that WNS-specific UV fluorescence [22] disappears within two to three weeks of euthermy.

Quantified per-unit wing membrane area fungal load correlates with disease intensity measured as the number of WNS lesions detected under UV transillumination [11]. It has been hypothesised that *P*. *destructans* hyphae are more likely to invade deeper skin layers and produce WNS lesions with heavy fungal growth on bat wings [11]. In fact, our data documents progression of fungal infection to WNS pathognomonic single and multiple cupping erosions in response to one- and several-orders-of-magnitude higher wing membrane fungal loads, respectively, compared with the early stage of infection characterised by skin surface colonisation (cf. Fig 7A). For this chronic skin infection, severity-grading scores were understandably higher in bats with more extensive UV fluorescence (Fig 8). The UV fluorescence associated with WNS is caused by riboflavin, a fungal secondary metabolite, that accumulates in skin lesions and results in cytotoxic damage of adjacent tissues [23] (as reflected by the weighted cumulative WNS pathology score). All three diagnostic methods (i.e. qPCR, UV transillumination and histology), when used in a quantitative or semi-quantitative manner, proved useful for discrimination between colonisation of bats exposed to the pathogen only and those with the invasive skin infection known as WNS.

Comparative findings from Palearctic and Nearctic bats infected with *P*. *destructans* show differences in prevalence, fungal load and qualitative [11] and semi-quantitative histopathology (this study). To gain greater insight into WNS severity, it is imperative that complex data are collected on infection intensity in each bat. Our proposed biopsy-based grading system for WNS is suitable for this purpose as histopathology reveals degree of invasiveness in terms of skin layers invaded, along with associated damage; qPCR provides data on pathogen load, and image analysis of photographs taken under UV light quantifies total affected wing/body surface area. The ease of diagnostic scoring using our system allows open, effective WNS surveillance across Europe, as well as comparative studies across the Holarctic. The use of uniform criteria and training sessions in WNS histopathology can substantially improve consensus rate in diagnosis of the disease among pathologists and promote its universality. Undertaken alongside UV trans-illumination, which enables targeted biopsy, our WNS severity-grading system provides an attractive option for use with conservation-sensitive bat species.

## Supporting information

S1 Table Species sample sizes. *n*–number of individuals per species undergoing histopathological examination, WNS histo+–number of individuals confirmed positive for white-nose syndrome.
(PDF)

S2 Table Data on fungal load, ultra-violet fluorescence and histopathology in Holarctic bats. Fungal load and UV fluorescence are given on log_10_ scale per cm^2^ of wing area. Histopathology is reported as a binary code for index signs of grades corresponding to those listed in Methods and Table 2. NA–not available, CZ–Czech Republic, LV–Latvia, PL–Poland, RU–Russia, SI–Slovenia, histoSum–weighted cumulative WNS pathology score.
(TXT)
