# Supplementary material for: White-nose syndrome pathology grading in Nearctic and Palearctic bats
Source: PLoS One. 2017 Aug 2;12(8):e0180435. doi: 10.1371/journal.pone.0180435 (PMC5540284; doi:10.1371/journal.pone.0180435)
Supplement: S1 Table — n–number of individuals per species undergoing histopathological examination, WNS histo+–number of individuals confirmed positive for white-nose syndrome. (PDF) [file pone.0180435.s001.pdf]

S1 Table. Species sample sizes. *n* = number of individuals per species undergoing histopathological examination, WNS histo+ = number of individuals confirmed positive for white-nose syndrome.

Species	*n*	WNS histo+
Barbastella barbastellus	4	3
Eptesicus nilssonii	4	3
Eptesicus serotinus	1	0
Miniopterus schreibersii	6	1
Myotis alcathoe	7	0
Myotis bechsteinii	8	3
Myotis brandtii	3	1
Myotis dasycneme	19	13
Myotis daubentonii	21	10
Myotis emarginatus	8	6
Myotis lucifugus	10	10
Myotis myotis	69	50
Myotis nattereri	10	4
Myotis septentrionalis	7	7
Nyctalus noctula	8	0
Pipistrellus pipistrellus	2	0
Pipistrellus pygmaeus	2	0
Plecotus auritus	12	7
Plecotus austriacus	1	0
Rhinolophus euryale	1	1
Rhinolophus hipposideros	7	4
**Total**	**210**	**123**
